# Reviewing suitability of Essex Partnership University Foundation NHS Trust out of area locked rehab placements

**DOI:** 10.1192/bjo.2021.815

**Published:** 2021-06-18

**Authors:** Shereen Ali, Miland Karale, Shaimaa Aboelenin, Ayesha Ahmed, Dolly Adulesi, Emmanuel Adebayo

**Affiliations:** 1 Essex Partnership Universty NHS trust, Essex Partnership NHS founation trust; 2 Essex partnership NHS founation trust, Essex Partnership University trust; 3 Essex Partnership NHS trust

## Abstract

**Aims:**

To look at 14 EPUT out of area patient profiles, map their journey to the current locked rehab placements -To review the appropriateness of placement of 14 patients through reviewing whether the care provided is achieving the rehabilitation goals.

To look at patients’ needs and whether the local alternatives can provide the care

**Background:**

Rehabilitation services aim to help complex General Adult Mental health patients reintegrate in the community by promoting independent living skills. Some complex mental health patient's care needs mandate a specialist rehabilitation services. Currently there has been a nationwide shortage of local rehabilitation services. This resulted in placing complex needs patients out of area in locked rehabilitation hospitals and miles away from their local community connections. Families and local community team providers travel miles to keep in contact with their complex need persons. The NHS five year plan includes minimizing the current out of area placements and for local services to work together as per CQC recommendations to work together and bring those individuals closer to home.

**Method:**

We designed a tool and examined the electronic records for all 14 out of area placed patient profiles, mapping their clinical journey and reviewing whether the care provided is achieving the rehabilitation goals.

**Result:**

(N = 14), Patient profiles: 78.5% had residual symptoms (Psychotic symptoms 85%). Patient's Illness profile; treatment resistant with residual symptoms in 71.4% and 7% had comorbid illicit substance misuse, other illness profiles 21.4%. History of alcohol and illicit drug misuse was present in 78.5% and 45% of them were using illicit substances more than 5 years. .patients’ risk profile revealed 86.7% had history of non-compliance. Attempted suicide 21.4% has attempted suicide at list once in which 1/3 of them had more than one attempt. 64.3% Had positive history of offending behavior. All patients in the sample had history of violence 85.7% had risk of vulnerability and self-neglect, 28.5% has history of carrying weapons, 35.7 had a previous Custodial sentence. Average Duration of illness average 16.7 years, average distance from home was 149 miles though clozapine was considered in 92.8% only 35.7% of sample was on clozapine, and the other 64.3% were on combinations. Only 35.7% were on depot.

**Conclusion:**

There is a need for expert input for advice regarding complex Management of residual symptoms and rehabilitation needs in the community. Health and social care joint working is needed.

